# Transmission Parameters of the 2001 Foot and Mouth Epidemic in Great Britain

**DOI:** 10.1371/journal.pone.0000502

**Published:** 2007-06-06

**Authors:** Irina Chis Ster, Neil M. Ferguson

**Affiliations:** Department of Infectious Disease Epidemiology, Imperial College London, Norfolk Place, London, United Kingdom; National Institute for Communicable Diseases, South Africa

## Abstract

Despite intensive ongoing research, key aspects of the spatial-temporal evolution of the 2001 foot and mouth disease (FMD) epidemic in Great Britain (GB) remain unexplained. Here we develop a Markov Chain Monte Carlo (MCMC) method for estimating epidemiological parameters of the 2001 outbreak for a range of simple transmission models. We make the simplifying assumption that infectious farms were completely observed in 2001, equivalent to assuming that farms that were proactively culled but not diagnosed with FMD were not infectious, even if some were infected. We estimate how transmission parameters varied through time, highlighting the impact of the control measures on the progression of the epidemic. We demonstrate statistically significant evidence for assortative contact patterns between animals of the same species. Predictive risk maps of the transmission potential in different geographic areas of GB are presented for the fitted models.

## Introduction

The 2001 FMD epidemic in the UK had a substantial cost in human, animal health and economic terms (Alexandersen et al. [1], Kao [2]). Understanding the risk factors underlying the transmission dynamics of that epidemic and evaluating the effectiveness of the control measures are essential to minimise the scale and cost of any future outbreak. Epidemic modelling [3], [4], [5]–[7] proved critical to decision making about control policies which were (in some cases controversially) adopted to control the 2001 epidemic [8]–[10]. Modelling now has a ‘peace-time’ contingency planning role.

One weakness of the modelling studies undertaken in 2001 was the relatively ad-hoc nature of the parameter estimation methods employed. In their first paper, Ferguson et al. [4] used maximum likelihood methods to fit to the observed incidence time series, but did not attempt to fit to the spatio-temporal pattern of spread. In their later work, the same authors developed a more robust method for estimating species-specific susceptibility and infectiousness parameters and spatial kernel parameters (see Supplementary Information to [3]), but at the time the statistical basis for the methods developed was lacking. In retrospect, the methods developed turned out to be closely related to those developed during the SARS epidemic by Wallinga and Teunis [11], although the earlier work incorporated population denominator data to allow for spatial- and species-based heterogeneity in disease transmission. Nevertheless, the methods employed had the limitation of not being fully parametric, meaning they could not be extended to fit arbitrary transmission models to the observed data. Keeling et al. [5] used maximum likelihood methods to estimate transmission parameters, but it was also supplemented by more ad hoc least-squares matching to regional incidence time series.

Therefore there remains a need to develop rigorous modern statistical approaches for parameter estimation of non-linear models for the 2001 FMD outbreak. Bayesian Markov Chain Monte Carlo (MCMC) techniques are the best established such methods and have been successfully employed in the analysis of a range of spatiotemporal outbreak data in the past [12]–[14], as well as to purely temporal incidence data [15], [16]. Here we develop MCMC-based inference models for the 2001 FMD epidemic in GB. The models examine: the extent to which transmission was spatially localised and the temporal variation in transmission, species-specific variation in susceptibility, infectiousness and heterogeneity in contact rates between and within species.

## Methods

### Data

We take the farm as the unit of our study and ignore the possible impact of within-farm epidemic dynamics. Thus we implicitly assume disease spread within a farm is so rapid as to be practically instantaneous, with all animals on a farm becoming infectious at the same time.

**Figure 1:**
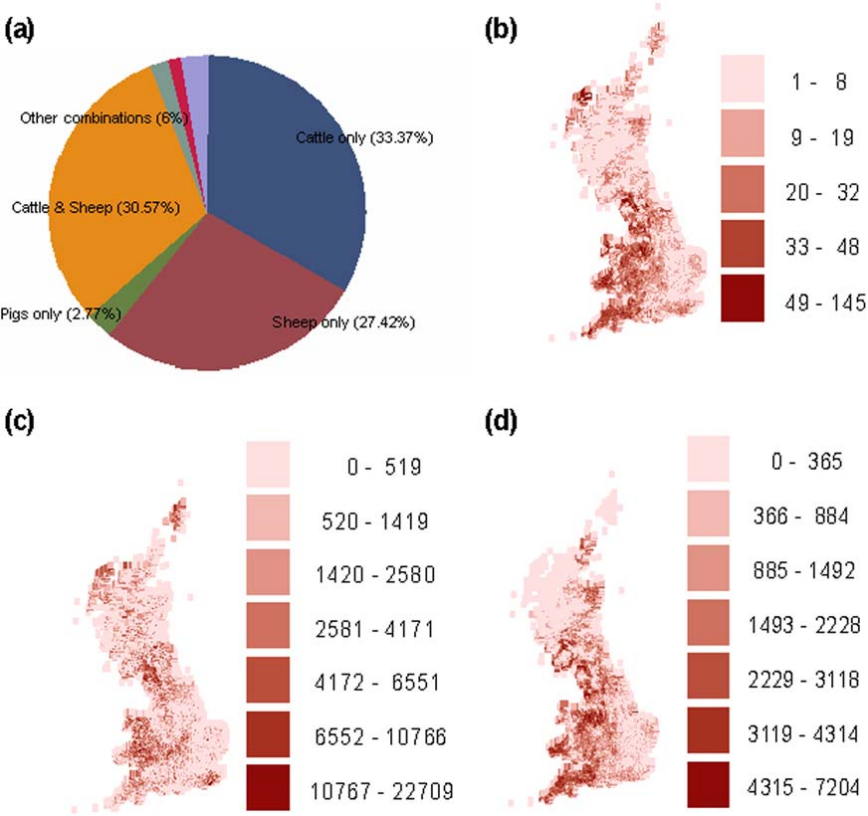
GB livestock population in 2000. (a) GB livestock farms partitioned according to the animal species kept. (b) Map of density of livestock farms. The number of livestock farms in each 5×5 km is plotted. (c) As (b) but plotting numbers of sheep kept per 5×5 km square. (d) As (c) for cattle.

Our data consists of information on all the farms in the UK listed in the 2000 agricultural census [see http://www.defra.gov.uk/footandmouth/cases/index.htm ]. There were a total of 134,986 farms listed in that dataset and uniquely identified by their County/Parish/Holding (CPH) number. Their spatial coordinates are provided together with the number of animals by species within each farm. A partition of all GB farms according to the animal types represented is shown in Figure 1a. Their geographical distribution is represented in Figure 1b as the number of farms per 5×5 km. Notice the high density areas in the North West (Cumbria), South West (Devon), Wales and Scotland where the main epidemic foci developed. There is also an area of high density in the Shetland Islands corresponding to very small crofter smallholdings. Figure 1c and d show the numbers of sheep and cattle kept per 5×5 km square.

During the 2001 FMD outbreak, a total of 2026 *infected premises* (IPs) were recorded – farms where FMD was diagnosed, and which were subsequently culled. The IP dataset contains, for each farm, the estimated date of infection (determined by a clinical evaluation of the age of lesions on affected animals), and the dates of disease reporting, confirmation and culling.

A total of 7457 other (non-IP) farms were also culled – mostly as *contiguous premises* (CPs, about 3103) or *dangerous contacts* (DCs, about 1287), but some under other local culling policies used in Cumbria and Scotland. For instance about 1846 (79%) out of a total of 2342 sheep farms in Cumbria had all sheep culled under the “local 3 km radial sheep cull” policy adopted there. Some of the farms (about 30) were recorded both as DCs and CPs. Multiple records per farm were often found in the disease control management system dataset, and it was often unclear whether this was due to data entry errors or as a result of sequential species-specific culls on the same farm. In our analysis we therefore considered the whole farm to be culled at the last recorded date of culling.

The most frequent species are cattle and sheep (see Figure 1a). There are less than 3% farms with pigs only and only 10 farms with just pigs were diagnosed as IPs in 2001 (less than 1% of all the IPs). This indicates a-priori that pigs contributed far less to the 2001 outbreak than many other FMD outbreaks (despite their high levels of shedding [1], [17]), and we therefore decided to discard pigs-only farms from the current study to simplify the analysis. The Sensitivity Analysis section shows that this simplification does not significantly affect estimates of other epidemiological parameters. We discarded another three IPs due to missing information or possible mistakes regarding their location or number of animals, leaving a total of 2013 IPs in our analysed dataset.

### Model formulation

We model the epidemic as a space-time survival process [18]. The total observation time *T* is the 240 days between 7^th^ February and 5^th^ October 2001. Each farm *i* at the location (*x_i_*, *y_i_*) is associated with an infection time *t_i_* (if infected), a removal time *r_i_* (if slaughtered) and two integers 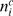 and 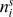 representing, respectively, the number of cattle and sheep on the farm. *S_c_* and *S_s_* represent per-capita cattle and sheep susceptibility, respectively, while *I_c_* and *I_s_* represent per capita cattle and sheep infectivity. The susceptibility is a relative measure of animal sensitivity to the disease whereas infectivity represents the infectious risk posed by an animal to others. We use a continuous kernel to describe how the probability of contact between farms scaled with distance. Transmission is naturally assumed to decrease with the distance between farms according to the power law
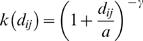
(1)where *d_ij_* represents the Euclidian distance between the infected farm *i* and the susceptible farm *j*. The parameters *a* (kernel offset) and γ (kernel power) are to be estimated. The kernel captures all forms of movement and contact between farms and as such, the use of a simple 2 parameter function is inevitably a highly simplified representation of the true complexity of inter-farm contacts. We examined other functional forms for the kernel (such as those used in some other analyses [19]) but the resulting model fits were much poorer than found using the power-law kernel above.

Given the susceptibility and infectiousness parameters and the kernel, the infection hazard from an infected farm *j* to a susceptible farm *i* is then quantified by
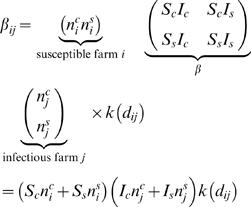
(2)This model is over-specified as stated, so we arbitrarily assume *S_s_* = 1 throughout, meaning *S_c_* represents the ratio of cattle-to-sheep susceptibility. For a constant (distance-independent) kernel this is just a mass-action closed epidemic model with heterogeneous susceptibility and infectiousness. This model assumes susceptibility and infectiousness parameters scale linearly with the number of animals of different species on the farm, a relatively strong assumption imposed for model parsimony reasons. The mixing matrix embedded in (2) quantifies the 4 species-specific mixing rates between animals on different farms: cattle-to-cattle (*S_c_I_c_*) sheep-to-cattle (*S_c_I_s_*), cattle-to-sheep (*S_s_I_c_*) and sheep-to-sheep (*S_s_I_s_*). This model formulation is identical to that used by Keeling et al. [5], except for the functional form of kernel used.

The *force of infection* on a *susceptible* farm *i* at time *t* depends on the whole history of events and is just
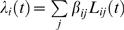
(3)where
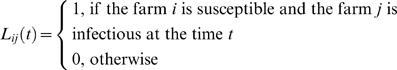
(4)By default, we assume a latent period of 1 day (latency is represented within the function *L*); i.e. farms are infectious the day after they are infected. However, we test the sensitivity of our estimates to the assumption by also examining latent periods of 2 and 3 days.

The probability density function that farm *i* is infected at time *t* is then given by
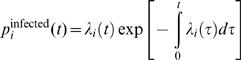
Hence, the contribution that a farm *i*, observed to be infected at time *t*, makes to the log likelihood is just:
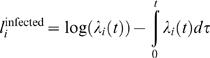
(5)A farm which is not infected contributes to the overall likelihood the probability that it escapes infection during the observation period, i.e. until the time it is culled (*r_i_*) or for the duration of the epidemic *T*, whichever is shorter. Its contribution to the log likelihood is therefore
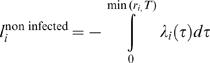
(6)The total log likelihood of the model can be written as
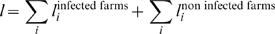
(7)We then extend the simple model above by introducing an additional parameter to understand to what extent the transmission within species is altered by between species transmission. The parameter ρ quantifies the degree to which mixing between species is assortative – with ρ<1 representing assortative mixing and ρ>1 disassortative mixing. The interaction model still assumes constant parameters with respect to time along the whole observation period *T*. The mixing matrix defined in equation (2) becomes
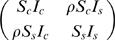
(8)where we again fix *S_s_* to be 1 to avoid model over specification. The force of infection (3) and model log likelihood equation (7) change accordingly.

Assuming transmission parameters were constant in time throughout the epidemic is obviously a crude simplification. However, allowing infectivity to vary continuously in time results in an over-specified model and problems of parameter identification and confounding. We therefore examined two sets of models in which changes in transmission parameter were restricted to 2 significant points in time denoted by *T_cut_*, namely 23^rd^ February (when the national ban on animal movements was introduced) and 31^st^ March (when control measures were intensified and the so called 24/48 hour IP/CP culling policy was introduced). Models were respectively fitted to the individual case data from the start of the epidemic (conditioning on the first infection) or from after 23rd February (conditioning on the 54 farms that were already infected by that date). A detailed history of the epidemic is given by Kao [9].

We separately fitted model variants which assumed a discrete change in parameters on 23^rd^ February and on 31^st^ March. Confounding meant that only a very limited number of parameters could be varied in time, so we examined the effect of varying infectiousness and kernel parameters separately. We fitted four separate time-varying model variants: (i) varying the cattle infectivity by a factor and keeping sheep infectivity constant through time (Cattle Infectivity model); (ii) varying sheep infectivity by a factor but not cattle infectivity (Sheep Infectivity model); (iii) varying both cattle and sheep infectivity by the same ratio (Cattle & Sheep Infectivity model); (iv) varying the kernel parameters (Time Varying Kernel model). For the last model variant we also fitted a version which includes non-assortative mixing between species (see equation (8)). Hence the most general mathematical expression of the transmission model is:
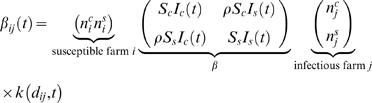
(9)where
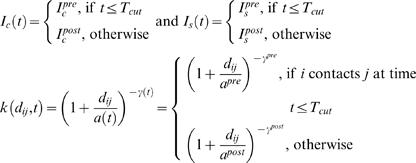
(10)The scripts *pre* and *post* are self-explanatory for time varying parameters. When fitting models with time varying infectivity parameters we actually fit *I^post^* and the ratio μ = *I^pre^/I^post^* we called infectivity factor. This is a within species ratio, a parameter directly fitted by the models, unlike the between species infectivity ratio additionally calculated as explained later in the text (see Parameter estimates section).

Note that all models above treat the epidemic as fully observed, i.e. infection times are assumed to be known (when in fact only estimated infection times are known – see Sensitivity Analysis section), and only IPs are assumed to be infectious.

### Statistical inference and model comparison

We adopt a Bayesian framework for statistical inference and use MCMC methods for fitting the model to individual case data. This is not strictly necessary, given our simplifying assumption that the epidemic was completely observed, but it provides a more consistent and robust framework within which to relax that assumption in future work.

We obtained parameter estimates and equal-tailed 95% credible intervals from the marginal posterior distributions of the fitted parameters. For the basic model for instance we estimated the relative cattle susceptibility, *S_c_*, two infectivity parameters (*I_c_*(*t*)≡*I_c_* and *I_s_*(*t*)≡*I_s_* for all *t*) and two kernel parameters (γ(*t*)≡γ*^post^*≡γ*^pre^*≡γ and *a*(*t*)≡*a^post^*≡*a^pre^*≡*a* for all *t*).

We used the posterior mean deviance as a Bayesian measure of fit or model adequacy as defined by Spiegelhalter et al. [20]. The posterior density deviance is defined as:
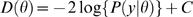
where log{*P*(*y*|**θ**)} is the log-likelihood function for the observed data vector *y* given the parameter vector **θ** and *C* is a constant which does not need to be known for model-comparison purposes (being a function of the data alone). The smaller the mean posterior deviance, the better the corresponding model fits the data.

If the posterior deviance distributions for two different models overlap significantly, it is necessary to use additional criteria to compare model fit – namely a comparison of the relative complexity of the models. The Deviance Information Criterion (DIC) is perhaps the most general of such methods, being a generalisation of the Akaike information criterion for Bayesian hierarchical models [20]. We define the complexity of a model by its effective number of parameters, *p_D_*, defined as
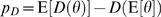
where E[ ] represents taking expectations (the posterior average). The DIC is then defined as
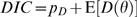



A lower value of DIC corresponds to a better model. This criterion offers flexibility for comparing non-nested models [20] and it is straightforwardly computed within an MCMC algorithm.

We applied the classic random walk Metropolis Hastings algorithm [21], [22] and a block-sampling of parameters due to the computationally expensive form of the likelihood [23], [24]. A log scale has been used for sampling as the parameters were all positive definite and were expected to potentially vary by orders of magnitude. However, linear scale sampling yielded similar results. The convergence of the chains was also very much improved (see Robert [25] for more on perfect sampling and reparameterization issues) compared with sampling on a linear scale. The model was coded in C and parallelized using OpenMP 2.0.

The MCMC sampler was allowed to equilibrate with convergence being evaluated visually from the likelihood and parameter traces. For the simpler models, 5,000 iterations were sufficient for equilibration, while this increased to 20,000 for the most complex models. Also, using log scale sampling, we verified that the chains were able to converge even if started with initial parameter values far from the final posterior mean values. Posterior distributions were estimated from 100,000 iterations. The rate of the acceptance varies from model to model. For the baseline model we achieved a 25% rate of acceptance and for the most complex model (8 parameters), a rate of approx 10%. These values compare well with the “golden” acceptance rate for Random Walk Metropolis Hastings of 23% (Roberts [26]).

We did not encounter common problems in MCMC estimation like slow convergence and slow mixing (O'Neill [27]). There were some correlations between parameters, mostly having biological explanations (cattle and sheep infectivity for instance), but a careful parameterization lowers them. We verified parameter estimates were not dependent on parameterization choices – e.g. no difference was seen whether we fitted species infectivity individually, or just fitted sheep infectivity and then the ratio of cattle-to-sheep infectivity.

## Results

### Parameter estimates

**Figure 2:**
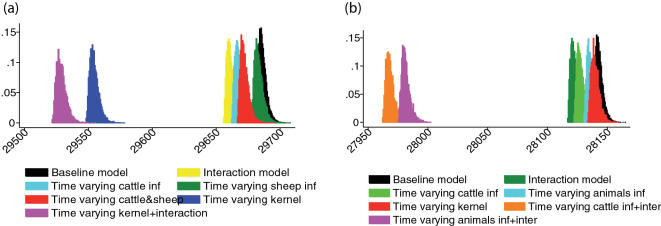
Posterior deviances. (a) Models conditioned on the first infection with parameters change at 23^rd^ February. (b) Models conditioned on 23^rd^ February with parameters change at 31^st^ March.

Table 1 lists the parameter estimates we obtained for a set of fitted models conditioned only on the first infection whereas Table 2 presents the estimates for models conditioned on infections occurring up to 23^rd^ February. The posterior deviances for each set of models are plotted in Figure 2a and Figure 2b, respectively. Figure 2a illustrates some clear conclusions. Of the two models without time variation in parameters, the interaction model fits significantly better than the baseline model without heterogeneous mixing between species. However, fitting the interaction model broadened the credible intervals of the infectivity parameter estimates (Table 1), indicating (unsurprisingly) slight confounding between the 4 infectivity and susceptibility parameters.

**Table 1:** Models conditioned on the first infection and 23^rd^ February time changing point if applicable. (95% equal-tailed credible intervals).

Model	Baseline model	Interaction model	Cattle Infectivity model	Sheep Infectivity model	Cattle & Sheep Infectivity model	Time Varying Kernel model	Time Varying Kernel + Interaction model
**Estimates**	**Mean (SD)**						
	**Equal tailed 95% Credible Interval**						
**Susceptibility Ratio** 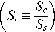	6.86 (0.44) (6.04,7.76)	5.95 (0.57) (4.93, 7.09)	6.83 (0.44) (6.02, 7.76)	6.81 (0.46) (5.99, 7.77)	6.80 (0.45) (5.97, 7.73)	6.74 (0.45) (5.97, 7.73)	5.71 (0.55) (4.62, 6.78)
**Cattle Inf (** ***I_c_*** **) (×10^8^) (Overall or Post 23 Feb)**	8.60 (1.06) (6.69, 10.8)	11.9 (1.88) (8.68, 15.90)	8.29 (0.99) (6.47, 10.40)	8.55 (1.07) (6.57, 10.80)	8.29 (0.99) (6.63, 10.50)	8.31 (0.98) (6.63, 10.50)	11.7 (1.89) (8.64, 16.00)
**Sheep Inf (** ***I_s_*** **) (×10^8^) (Overall or Post 23 Feb)**	1.43 (0.18) (1.11, 1.82)	2.16 (0.31) (1.65, 2.82)	1.37(0.17) (1.05, 1.71)	2.47 (0.55) (6.63, 10.50)	1.34 (0.17) (1.05, 1.71)	1.30 (0.17) (1.04, 1.7)	2.00 (0.28) (1.52, 2.62)
**Assortativity Factor (ρ)**		0.47 (0.075) (0.33, 0.63)					0.45 (0.075) (0.31, 0.61)
**Inf factor (μ) Pre∶Post 23^rd^ Feb**			3.17 (0.58) (2.11, 4.38)	2.47 (0.55) (1.54, 3.71)	2.11 (0.29) (1.57, 2.70)		
**Kernel power (Pre 23^rd^ Feb)**						1.72 (0.098) (1.54, 1.93)	1.69 (0.10) (1.51, 1.92)
**Kernel offset (Pre 23^rd^ Feb)**						690 (160) (414, 1066)	694 (166) (376, 1035)
**Kernel power (Overall or Post 23^rd^ Feb)**	2.58 (0.05) (2.49, 2.67)	2.56 (0.05) (2.47, 2.66)	2.56 (0.05) (2.50, 2.68)	2.58 (0.05) (2.49, 2.68)	2.58 (0.05) (2.50, 2.67)	2.68 (0.05) (2.58, 2.78)	2.67 (0.05) (2.56, 2.77)
**Kernel offset (Overall or Post 23^rd^ Feb)**	1190 (104) (1006,1412)	1175 (106) (978, 1389)	1212 (102) (1030, 1432)	1203 (108) (1014, 1434)	1207 (97) (1029, 1407)	1329 (118) (1098, 1560)	1317 (116) (1103, 1151)
**Posterior deviance**	29687	29662	29668	29684	29672	29555	29529
**Complexity**	4.6	5.1	5.7	5.7	5.7	6.5	7.2
**DIC**	29691	29667	29674	29689	29678	29561	29536

**Table 2:** Models conditioned on 23^rd^ February and 31^st^ March time changing point if applicable.

Models	Baseline model	Interact model	Cattle Infectivity model	Cattle & Sheep Infectivity model	Cattle &Sheep Infectivity +Inter model	Cattle Infectivity + Interaction model	Time Varying Kernel model
**Estimates**	**Mean (SD)**						
	**Equal tailed 95% Credible Interval**						
**Susceptibility Ratio** 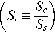	7.24 (0.47) (6.37, 8.21)	6.36 (0.57) (5.25, 7.50)	7.35 (0.49) (6.46, 8.34)	7.34 (0.49) (6.44, 8.35)	5.95 (0.54) (4.95, 7.03)	6.08 (0.6) (4.95, 7.30)	7.35 (0.54) (6,32, 8.49)
**Cattle Inf (** ***I_c_*** **) (×10^8^) (Overall or Post 31^st^ Mar)**	7.64 (1.0) (5.86, 9.75)	10.30(1.60) (7.55, 13.7)	8.9 (1.17) (6.8, 11.4)	8.41 (1.1) (6.46, 10.8)	12.1 (1.81) (9.02, 16.1)	13.5 (2.21) (9.73, 18.2)	7.81 (0.54) (6.32, 8.49)
**Sheep Inf (** ***I_s_*** **) (×10^8^) (Overall or Post 31^st^ Mar)**	1.31 (0.18) (0.98, 1.70)	1.93 (0.27) (1.45, 2.51)	1.32 (0.18) (1.01, 1.71)	1.43 (0.19) (1.07, 1.86)	2.24 (0.3) (1.71, 2.91)	2.27 (0.3) (1.71, 3)	1.33 (0.17) (1.04, 1.72)
**Assortativity Factor (ρ)**		0.49 (0.08) (0.34, 0.67)			0.51 (0.08) (0.36, 0.68)	0.45 (0.08) (0.3, 0.6)	
**Infectivity factor (μ) Pre∶Post 31^st^ March**			0.73 (0.05) (0.63, 0.83)	0.85 (0.04) (0.77, 0.92)	0.88 (0.04) (0.81, 0.97)	0.72 (0.05) (0.62, 0.82)	
**Kernel power (Pre 31^st^ Mch)**							2.61 (0.06) (2.50, 2.73)
**Kernel offset (Pre 31^st^ March)**							1216 (115) (1015, 1464)
**Kernel power (Overall or Post 31^st^ March)**	2.68 (0.06) (2.58, 2.8)	2.67 (0.06) (2.57, 2.78)	2.69 (0.06) (2.58, 2.7)	2.68 (0.05) (2.58, 2.7)	2.67 (0.05) (2.57, 2.78)	2.67 (0.05) (2.57, 2.78)	2.74 (0.07) (2.62, 2.89)
**Kernel offset (Overall or Post 31^st^ March)**	1344 (131) (1114, 1617)	1334 (123) (1116,1601)	1353 (128) (1114, 1629)	1339 (123) (1117, 1630)	1308 (114) (1092, 1543)	1312 (120) (1078, 1552)	1437 (130) (1192, 1709)
**Posterior deviance**	28144	28122	28128	28136	27981	27968	28140
**Complexity**	4.6	4.9	5.6	5.4	6.3	6.5	6.9
**DIC**	28149	28128	28134	28142	27987	27975	28148

**Figure 3:**
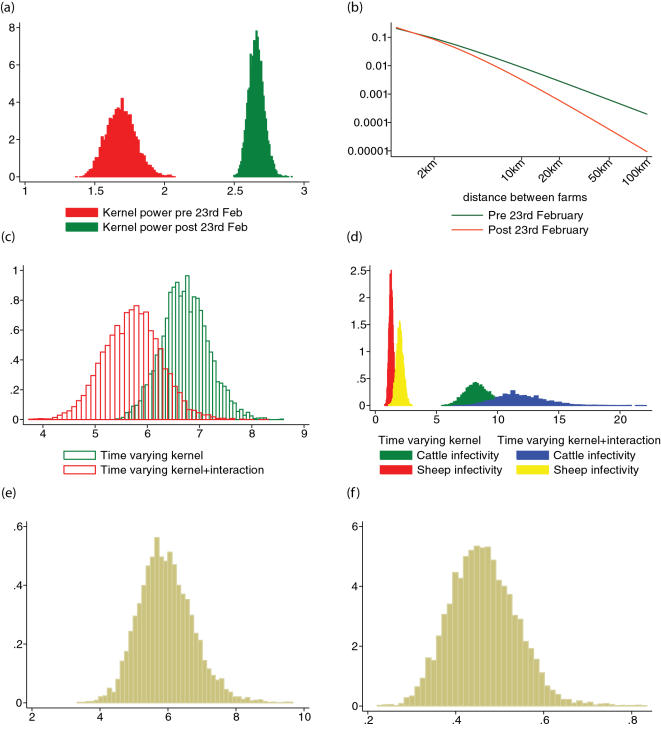
Posterior densities for the estimated parameters from the most complex models conditioned on the first infection allowing for parameter changes on 23^rd^ Feb and interaction. (a) Spatial kernel powers γ pre and post 23^rd^ February. (b) Pre and post 23^rd^ February estimated kernels, a log-log scale plot. (c) Susceptibility ratios cattle∶sheep and (d) Animals infectivity parameters as modified by interaction in time varying kernel model. (e) Infectivity ratio cattle∶sheep as calculated from the most complex model. (f) Assortativity parameter.

Of the models which allowed infectivity to vary on 23^rd^ February, allowing only cattle infectivity variation gave a slightly better fit than varying sheep infectivity or both. However, of the models with parameters which vary on 23^rd^ February, the model variants which allow the 2 kernel parameters to vary at that time point fit substantially better (by both deviance and DIC criteria, see Table 1) than those which just allow a species-specific variation in infectivity. This is encouraging for the inference procedure, as the main control measure initiated on that date was the banning of all animal movements – which would be expected to have a major impact on the spatial component of the transmission. We found strong evidence for the kernel decaying much more rapidly with distance after 23^rd^ February, with γ*^pre^* = 1.72 (1.54, 1.93) before 23^rd^ February and γ*^post^* = 2.68 (2.58, 2.78) after that date (Figure 3a and Figure 3b). The parameter estimates are less precise before 23^rd^ February (Table 1) due to the relatively small number of IPs (about 57) before that date.

Looking at the most complex model (namely the interaction model with time varying kernel), cattle were estimated to be 5.7-fold (4.6, 6.8) more susceptible than sheep (see Figure 3c and Table 1). Rather than mentioning animals' specific infectivity (see Figure 3d and Table 1), it is more informative to comment on the cattle∶sheep infectivity ratio parameter for the most complex fit (this ratio does dot appear in the tables as it is not a model parameter). We calculated it within the MCMC algorithm as the ratio of the two species infectiousness for each sampled parameter point. The most complex model suggests that cattle are 5.95-fold (4.54, 7.63) more infectious than sheep (Figure 3e).

The parameter quantifying assortativity in mixing was estimated at ρ = 0.45 (0.31, 0.61) – well below 1, the level at which mixing between species is random (Figure 3f). By comparison with the model with a time varying kernel but random mixing between species, the effect of heterogeneous mixing between species modified the between-species transmission as given by matrix ^(1.9)^ as indicated below.
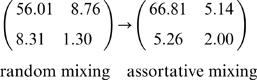
(11)Cattle-to-cattle and sheep-to-sheep transmission is higher (by 19% and 54% respectively) for the model with non-random mixing, whereas the sheep-to-cattle and cattle-to-sheep transmissions dropped by 41% and 37 % respectively.

Conditioned on 23^rd^ February, 7 model variants have been considered (Table 2 and Figure 2b). We examined the baseline and interaction models (no change in parameters over time), allowing cattle infectivity to vary on 31^st^ March and both cattle and sheep infectivity to vary by the same factor after 31^st^ March (with and without heterogeneity in mixing) and allowing both kernel parameters to vary on 31^st^ March.

Unsurprisingly, the kernel parameters were not significantly different if allowed to be different before and after 31^st^ March, neither did this model prove to be the best fit. Overall, while the variations in mean deviance (Figure 2b) seen between model variants were much smaller than for the models conditioned on the first infection (Figure 2a), the interaction model allowing for time varying cattle infectivity gave the most adequate fit (measured by both mean deviance and DIC, see Table 2).

We cannot statistically compare the two sets of models in Table 1 and Table 2, as the data used are different for the two cases. However, the parameter estimates from the best-fitting models of each table are largely consistent. Each post-23^rd^ February estimated value from the best-fit model in Table 1 is included in the corresponding pre-31^st^ March 95% credible interval of the best fit model in Table 2 (and vice-versa).

The most important message from the second set of models is that all models with cattle time varying infectivity (best fit) indicated higher values of infectivity after 31^st^ March than before (μ = 0.73 (0.63, 0.83)) (Table 2). This may seem paradoxical but reflects the fact that while culling (the effect of which is explicitly included in the input data) dramatically reduced case incidence in April, from May to September 2001, case incidence maintained itself at a low level – but almost entirely within cattle farms. This increase in cattle infectivity may therefore really reflect the impact of reduced biosecurity and/or increased non-compliance with movement controls.

### Risk maps

**Figure 4:**
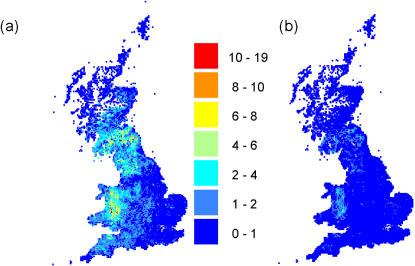
Maps of transmission risk (potential *R*_0_) before 23^rd^ February (a) and after (b) as predicted from the interaction model with time varying kernel conditioned on the first infection (Table 1 and text).

**Figure 5:**
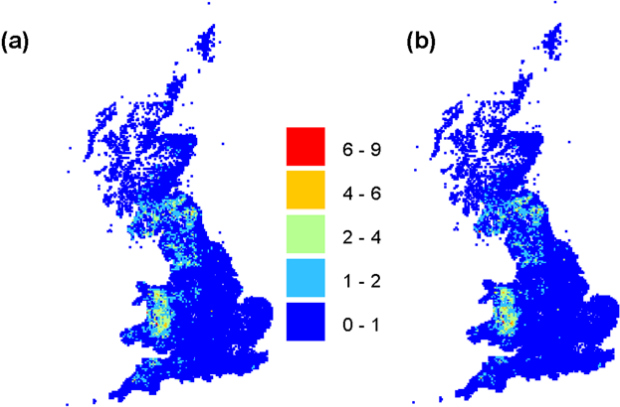
Transmission risk map (a) before 31^st^ March and (b) after 31^st^ March calculated from the interaction model with time varying cattle infectivity conditioned on 23^rd^ February (Table 2 and text).

It is informative to examine what our parameter estimates imply in terms of geographic variation in transmission potential. Given the parameter estimates for each model, we can define the relative risk of transmission an infectious farm *j* would pose to all susceptible farms in the country *r_j_*:
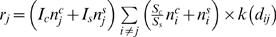
(12)This quantity multiplied by the average duration of infectiousness of a farm (time from end of latency to culling) gives the reproduction number *R_0j_* of the farm *j*. We divided the UK into 5 km squares and then calculated the average transmission risk of all farms in each square (local *R*_0_). Figure 4 shows how geographic risk changed before and after 23^rd^ February for our best fit model conditioned on the first infection. The kernel shape has a major influence on the average risk distribution throughout the country. Figure 5 shows the corresponding risk maps for the estimates inferred from our best fit model conditioned on 23^rd^ February. A slightly higher risk is predicted after 31^st^ March by the model conditioned on 23^rd^ February due to the increase in the cattle infectivity after this date. The risk estimates after 23^rd^ February from the first set of models appear consistent with those obtained from the models conditioned on 23^rd^ February, though a rigorous statistical comparison is not appropriate.

### Infections in proactively culled farms

We have made the strong assumption for this study that the only infected farms during the 2001 epidemic were the reported IPs, and hence that any farms which were infected but culled before clinical diagnosis were not responsible for causing any infections. It is therefore interesting to calculate how many of the proactively culled farms our model predicts might have been infected (but, by definition, not diagnosed).

To calculate the probability *p_i_* that a particular proactively culled farm *i* was infected, we need to adjust the infection hazard by the probability that the farm would have not been reported as a clinical case before its culling date 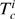. From the outbreak data, we calculate the probability density of the time from infection to report for reported IPs and hence the cumulative probability distribution of the time from infection to report, denoted by *F*. Then, with λ*_i_*(*t*) being the force of infection on a proactively culled farm *i* at time *t* (from the best fit model conditioned on 23^rd^ February), the probability that that farm gets infected and escapes reporting between its potential infection time and culling time 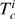 is
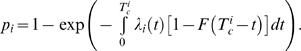
(13)We calculate the expected number of infections in different classes (e.g. DCs, CPs) of proactively culled farms culled within a particular time interval (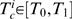). For instance, the expected number of CPs culled at the time 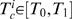 which are predicted to have been infected can be formally written as
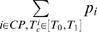
(14)This is a simplification, as in reality the delay from infection to report almost certainly depends on the size and species mix on a farm, but the result is nevertheless indicative of the expected level of infection in proactive culling. Also, at this stage, the calculations are made as if culling was a non-informative censoring process. This is a reasonable assumption for all proactively culled farms except for DCs (which by definition had been identified by veterinarian as having had a high risk of exposure) but our method may underestimate the infection rate. In calculating the infection to report delay distributions, we divided the epidemic after 23^rd^ February into 3 time periods: 23^rd^ February–31^st^ March, 31^st^ March–1^st^ May and 1^st^ May–5^th^ October. In these intervals a total of 1332, 4498 and 1627 farms were slaughtered, respectively. Our best fit model conditioned on 23^rd^ February predicts different infectivity regimes before and after 31^st^ March (see Parameter Estimates and Table 2) but we split further the second period of time due to different delays in reporting to culling. The infection to report delay is 8.6 and 8.8 days for the last two periods of time respectively but the infection to cull delay drops from 9.4 and 8.8 days respectively.

**Figure 6:**
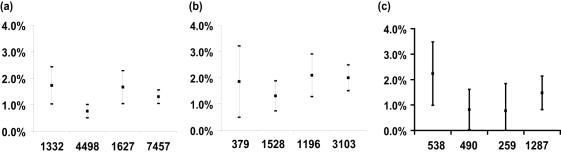
The estimated proportions of infections and their 95% CI for proactively culled farms. (a) Total number of proactively culled farms. (b) CPs culled farms (c) DCs culled farms. The first 3 figures on x-axis represent, in order, the numbers of farms culled within each epidemic stage we considered whereas the last figure represents the total number of culled farms after 23^rd^ Feb onwards.

Applying this approach to the interaction model with time varying cattle infectivity which conditioned on the 23^rd^ of February, we calculated the expected proportion of proactively culled farms which were infected. We estimate that approximately 1.3% (1%, 1.6%) of 7457 culled non-IP farms may have been infected – 97 in total (Figure 6a). Of the 1332 farms culled between 23^rd^ February and 31^st^ March, 1.7% (1%, 2.4%) may have been infected (23 farms). Of the 4498 farms culled between 31^st^ March and 1^st^ May, we estimate 0.7% (0.5%, 1%) were infected (34 farms). In the period 1^st^ May to 5^th^ October, we estimate that 1.6% (1%, 2.3%) of 1627 farms culled were infected (27 farms).

The proportion of CPs estimated to have been infected is 2% (1.5%, 2.5%), equating to 62 farms (Figure 6b). Over the whole epidemic, we estimated 1.5% (0.8%, 2.1%) of farms designated as DCs were infected (19 farms). This estimate (Figure 6c) does not allow for higher risk of infection implied by the veterinary judgement that led to those DCs being identified, which may mean that a higher proportion were in fact infected. If we assume that DCs were 3 times more likely to be infected due to their status than the model would predict, then the incidence of infection in DCs goes up accordingly, i.e. to 4.6% or 59 farms.

Farms culled neither as DCs or CPs (typically those culled under the 3 km and local sheep cull policies in the Cumbria, Dumfries and Galloway areas) had the lowest estimated rate of infection – a mere 0.5 % (0.2%, 0.8%) or 16 out of 3067 farms.

### Sensitivity Analysis

In this section we examine the sensitivity of our results to a number of factors: leaving pigs out of the analysis, possible errors in the estimated IP infection dates, and the assumed latent period.

To justify the simplification of the analysis by discarding the number of pigs in a farm, we present some more detailed statistics regarding this variable. We also fit the simplest model conditioned on the first infection including it into the analysis. Out of all reportedly infected farms 2026, 95% (approx. 1921 farms) of them have no pigs, 3% (about 80 farms) have less than 100 pigs and only 0.7% (about 14) have between 100 and 1000 pigs. There are only 4 big farms with 1110, 1400, 2000 and 4500 pigs from which only the last two farms are exclusively pig farms. We denote by 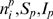 the number of pigs in farm *i*, pigs susceptibility and pigs infectivity respectively. The simplest model similar to (1.2) conditioned on the first infection has been fitted, reducing the number of parameters in the same manner.
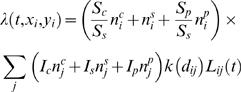
(15)In addition we estimated pig∶sheep susceptibility ratio and pig infectivity, assuming all parameters constant through time. We found that cattle∶sheep susceptibility ratio is 6.8 (sd 0.44), 95% CI (5.99, 7.72). Cattle and sheep infectivity estimates are 8.44 (sd 0.1) 95%CI (6.68, 10.6) and 1.38 (sd 0.18) 95% CI (1.08, 1.77) respectively (the last two are scaled by a factor of 10^8^). The kernel estimates are 2.58 (sd 0.05) 95%CI (2.49, 2.67) and 1186 (sd 100) 95% (999, 1392) for power and offset, respectively. We learned from this model that pigs are less susceptible than sheep, i.e. pigs∶sheep susceptibility ratio is estimated at 0.27 (sd 0.09) 95%CI (0.14, 0.48). Also, cattle are more infective than pigs by a factor of 1.89 (sd 0.49) 95%CI (1.18, 3.07), but pigs are more infective than sheep by a factor of 3.43 (sd 0.85) 95%CI (1.95, 5.27). A quick comparison with Table 1 shows parameter estimates for cattle and sheep are largely unaffected by ignoring the pig population, with none of the estimates from the two analyses being significantly different. We conclude that including pigs would not change the conclusions presented in Table 1 regarding cattle and sheep (given the very small number of IPs which had pigs) but it would decrease the power of the analysis and increase model complexity.

To understand to what extent our estimates are affected by the assumption that the infection dates have been accurately observed, we randomized the estimated infection dates by adding a Gaussian noise with zero mean and a standard deviation of 2 days. This is motivated by the substantial proportion in the observed standard deviation (73.5% less or equal than 2 days) of the distribution time from the estimated infection date to the report date of IPs. We then fitted the simplest model (conditioned on both first infection and 23^rd^ February) to 10 such randomised datasets. The average estimates across them are given in Table 3. They lie well within the confidence intervals we predicted in Table 1. The average cattle∶sheep infectivity ratio is also very close to the values estimated using the original data.

**Table 3:** Average estimates from 10 datasets with randomized infection times (see text).

Models	Baseline model (cond on the first infection)	Baseline model (cond on 23^rd^ Feb)	Cattle Infectivity + Interaction (cond on 23^rd^ Feb)
**Average estimates**	**Mean**		
**Susceptibility Ratio** 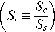	6.94	7.23	6.24
**Cattle Inf (** ***I_c_*** **) (×10^8^) (Overall or Post 31^st^Mar)**	7.15	6.69	11.58
**Sheep Inf (** ***I_s_*** **) (×10^8^) (Overall or Post 31^st^Mar)**	1.27	1.19	2.02
**Cattle∶Sheep Infectivity (additionally calculated)**	5.68	5.64	
**Assortativity Factor (ρ)**			0.46
**Infectivity factor (μ) Pre∶Post 31^st^ March**			0.70
**Kernel power**	2.62	2.72	2.71
**Kernel offset**	1327	1471	1445

The average estimates across 10 randomized datasets using the most appropriate model conditioned on 23^rd^ February (i.e. cattle infectivity and interaction model) are also in Table 3. The values are within the 95%CI presented in Table 2. We assessed a sensitivity analysis for the estimated proportion of infections in proactively culled farms (see the previous section) with respect to infection times. Using the predicted parameters for each dataset, we calculated the average proportions across all of them, for each category of proactively culled farms. The average proportion of infections between DC farms is 1.37% (2%, 0.78% and 0.72% for each period of time, respectively). For CP farms, the same quantities evaluate to 1.9% with 1.8%, 1.3% and 1.98%, respectively. Overall proactively culled farms, we obtained an average percentage of 1.25% with 1.64%, 0.81% and 1.6% for each considered period of time. All the values are well within the 95%CIs predicted by the original data (see the previous section and Figure 6).

All the results presented above assume a fixed latent period of 1 day. We tested the sensitivity of parameter estimates to this assumption by examining latent periods of 2 and 3 days. Overall, we would expect infectiousness parameters to increase to compensate for the shorter infectious period, and thus slightly increased generation time (namely the mean time from infection of one case and the time of infection of the cases that case generates). Interestingly, however, it is the kernel parameter estimates which are altered as the latent period is varied with the kernel becoming slightly less local with increasing latent period. For two and three days latent period, pre 23^rd^ February, the values of γ dropped from 1.69 (Table 1) to 1.51 and 1.46 respectively. After this date the same parameter estimate dropped from 2.67 (Table 1) to 2.64 and 2.59 respectively. This may reflect the fact that increasing the latent period decreases the prevalence and therefore density of infectious farms, thereby increasing the expected mean distance over which infection events occur.

## Discussion

This paper has presented a statistical analysis of the spatiotemporal evolution of the 2001 foot and mouth outbreak in GB. Qualitatively, the results agree with those obtained by Keeling et al. [5] in identifying cattle as being the key species in the 2001 epidemic. Using the interaction model conditioned on 23^rd^ February with time varying cattle infectivity, we estimated that 88% of IPs between 23^rd^ Feb–31^st^ March were infected by cattle and only 12% by sheep. Sheep-to-sheep transmission only accounts for 3.1% of IPs in that period. After 31^st^ March (when we estimated that cattle infectivity increased slightly, see Table 2) 91% of the IPs were infected by cattle and 8.9% by sheep, with sheep-to-sheep transmission accounting for only 2.3% of infections. While these levels of transmission from sheep farms are even smaller than previously thought [5], they are consistent with the results of experimental analysis [28] which indicate sheep cannot sustain an epidemic of the Pan O Asian strain of FMD virus.

Allowing for non-random mixing between species indicates contacts between farms are assortative on the basis of species composition of the farm; i.e. like species mix with like. This agrees with intuition about the nature of farming practices (e.g. sharing of personnel and equipment is likely to be more common if 2 farms have the same livestock species). The implications of the moderate degree of assortativity we found for control measures remains to be explored.

We did not use data collected during the epidemic on traced contacts between farms to fix the spatial kernel function in our analysis, since in the final version of the FMD epidemic data warehouse [http://www.defra.gov.uk/footandmouth/cases/index.htm] very few of the contacts apparently identified early in the epidemic remain confirmed. Also we shared the concern of earlier work that the distribution of contact distances in traced contacts may well be biased [3]. We therefore estimated the kernel function, using an offset power-law functional form. The higher value of the kernel power parameter we estimated after 23^rd^ February (2.67 vs. 1.70 before – Figure 3a) is consistent with the expected dramatic shortening in the typical contact distance following the national movement ban. This localized spread together with the higher estimated level of infectivity in cattle after 31^st^ March explains the long tail of the epidemic seen in 2001.

In estimating the transmission risk between farms, we assumed a dependence on the Euclidian distance between them. In reality, other metrics (e.g. the time required to travel between two farms) might be more reasonable, and should be examined in future work. We also did not include information on landscape (e.g. height above sea-level, location of rivers, trees etc).

The estimated risk maps (Figure 4 and Figure 5) match the areas of the country where highest case incidence rates were seen – with the notable exception of Wales. The discrepancy between the high predicted risk in Wales and the small number of cases observed may reflect inaccuracies in the input data set - Keeling et al. [5] reduced farm-level sheep population numbers by 30% in Wales and obtained a better geographic match to the data (Matt Keeling, personal communication). However, the discrepancy may also reflect model inadequacy. We have not here allowed for other farm-level risk factors, such as the farm fragmentation index considered by Ferguson et al. [3]. We have not explored more complex non-linear models of the dependence of susceptibility and infectiousness on the number of animals on a farm or relaxed our implicit assumption that contact rates between farms scale linearly with the local density of farms. All these assumptions are being relaxed in ongoing work.

The most important issue to be revised in future work is to allow for proactively culled farms which were not diagnosed as IPs to be potentially infected and infectious to other farms. This requires modification of the inference model used to allow for an arbitrary number of unobserved infections. The very low numbers of proactively culled farms we estimated as infected suggested that the effect of this model refinement may be limited. It should be noted though that these infection prevalence estimates are in part a result of the relatively non-local kernel estimated simultaneously. If kernel estimates change in a refined analysis – and if DCs were attributed a much higher risk of infection than estimated here due to their status – then it is possible that estimated infection rates in DCs and other proactively culled farms may increase somewhat.

However, even if these factors increased our estimated infection prevalence among proactively culled farms 5 fold (which seems unlikely from ongoing work), it would still mean that only a small proportion (<10%) of DCs and CPs culled were infected. This does not imply that proactive culling had no effect on the epidemic – as the largest expected effect of such culling is via the targeted depletion of susceptible animals. In this regard, proactive culling has the same epidemiological impact as vaccination. Future work will revisit past estimates of exactly how important such culling was for the control of the 2001 FMD epidemic.
